# RNA sequencing analyses in infants patients with coarctation of the aorta

**DOI:** 10.1186/s41065-021-00194-w

**Published:** 2021-08-23

**Authors:** Aijun Liu, Bin Li, Ming Yang, Yan Gu, Lihua Qi, Junwu Su

**Affiliations:** 1 Department of Pediatric Cardiac Surgery, Beijing Anzhen Hospital, Capital Medical University, An Zhen Rd, Beijing, 100029 China; 2 Department of Pediatric Cardiology, Beijing Anzhen Hospital, Capital Medical University, Beijing, 100029 China; 3 Department of Human Anatomy, Histology and Embryology, Peking University Health Science Center, No. 38, Xue Yuan Rd., Beijing, 100191 China

**Keywords:** Coarctation of the aorta (CoA), Infants, RNA sequencing, Smooth muscle cell differentiation

## Abstract

**Background:**

Coarctation of the aorta (CoA) is a serious innate heart disease. Although surgery results are generally good, some complications such as recoarctation and aortic aneurysm or persistent hypertension were serious threats to patient’s health. To better understand the pathology of CoA and its underlying molecular mechanism is particularly important for early diagnosis and preventing the occurrence of its complications. However, the mechanisms of CoA remain unclear, especially for infants.

**Methods:**

RNA sequencing (RNA-seq) was used to identify the differentially expressed genes (DEGs) in vascular tissues of 12 patients with CoA and 10 normal participants form 3- to 34-month-old infants. The characteristic of DEGs were validated by quantitative reverse transcription–polymerase chain reaction (qRT-PCR) and immunochemical staining (IHC) in vessels of patients with CoA and normal infants.

**Results:**

A total of 2491 DEGs with the false discovery rate less than 0.05(> 1.5-fold, *P* < 0.05 change) were identified, including 443 upregulated genes and 2048 downregulated genes. The Gene Ontology enrichment analysis showed that 26 out of the 2491 DEGs identified were associated with cardiovascular diseases. These 26 genes were mainly associated with extracellular matrix (ECM) and smooth muscle cells (SMCs) differentiation. Three DEGs, that is, CNN1 (calponin), α-actinin1 and myosin heavy chain 11 MYH11, were validated using qRT-PCR and Western blot analysis. In addition, immunochemical staining showed that calponin and MYH11 were highly expressed on the surface and in the deep layers of the thickened intima respectively.

**Conclusion:**

This study comprehensively characterized the CoA transcriptome. Migration of extracellular matrix (ECM) and smooth muscle cells (SMCs) to the subendothelial space may be the major characteristic of CoA in infants.

## Background

Coarctation of the aorta (CoA) is the most common cardiac abnormality, which accounts for 6–8% of all innate heart diseases [1]. It is usually located at the distal end of the ductus arteriosus insertion point and is characterized by narrowing of the aorta [2]. Early diagnosis and proper management of CoA have significant positive effects on the mortality rate associated with this disease [3]. However, the complications, such as paradoxical hypertension and aneurysm formation, still pose a serious threat to the health of patients with CoA [4, 5]. Revealing the molecular mechanisms underlying the pathogenesis of this condition may provide insights into the possible selection of the appropriate therapeutic measures for treating CoA, especially for improving the life quality of infants [6]. However, most existing reports mainly focused on surgical treatment and clinical management of postoperative complications [7]. So far, the underlying mechanisms of CoA remain unclear. Although Yoon and Kim showed that the proliferation of smooth muscle cells (SMCs) in the ductus leads to aortic isthmus (between the beginning of the left subclavian artery and the ductus insertion in the area of the descending aorta) [8], the exact molecular regulation mechanisms underlying the development of CoA need further verification, especially in infants. In this study, we examined novel various differentially expressed genes (DEGs) and canonical pathways with RNA-seq in 12 children with CoA and 10 control children. Novel DEGs were validated with immunohistochemical (IHC) analysis and quantitative reverse transcription–polymerase chain reaction (qRT-PCR). The study provided valuable information on the key genes expressed in infants with CoA and put forward new ideas regarding the molecular mechanisms and potential signal regulation in the aorta coarctation of infants.

## Methods

### Patients and samples

**Fig. 1:**
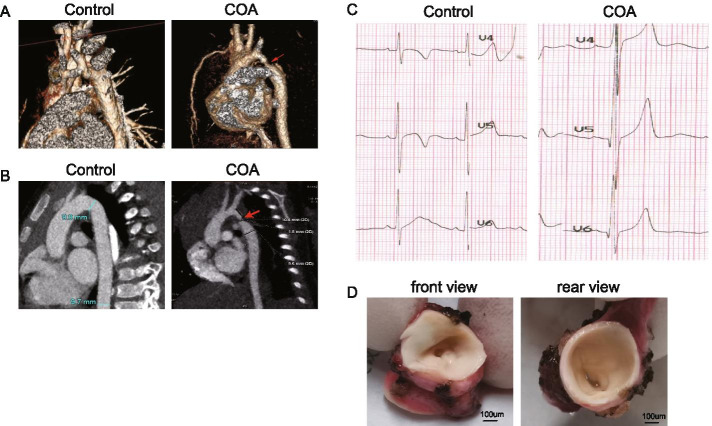
Diagnosis of CoA using computed tomography and electrocardiogram. **A** Three-dimensional reconstruction imaging of computed tomographic angiography showed control and CoA aortas. The arrow shows the site of discrete narrowing. **B** Two-dimensional sagittal reconstruction imaging of control and coarctation aortas. The arrow shows the site of discrete narrowing at the level of the aortic isthmus. **C** Electrocardiogram imaging of control and CoA aortas. **D** Representative macroscopic observations of the site of discrete narrowing

Data of 12 patients with CoA admitted to Beijing Anzhen Hospital, Capital Medical University, for surgical treatment of isolated aortic coarctation in 2019 were collected. Patients with aortic atresia, hypoplasia of aortic arch, ventricular septal defect aortic stenosis, left ventricular hypoplasia, mitral stenosis, history of previous aortic arch operation, or supravalvar mitral ring were excluded. Further, 10 control participants were recruited for the screening and identification of possible genes associated with CoA. The patients’ ages ranged from 3 to 34 months. The weight of the patients ranged from 3.5 kg to 12.5 kg (Table 1). The CoA and control groups were matched for sex, age and body weight. The criteria of surgery for CoA was a peak-to-peak gradient ≥ 20 mmHg diagnosed using transthoracic echocardiography or the narrowing of the isthmus greater than 40% of the diameter of the ascending aorta diagnosed using computed tomography angiography (CTA) [9] (Fig. 1A-C). The tissue at the aortic isthmus was resected and collected (Fig. 1D). Since tissues at the juxtaductal aorta could not be obtained due to ethical reasons. Tissues from other sites of the aorta were collected as controls. Due to the limited number, 10 control (normal) samples were collected from patients with anomalous left coronary artery from the pulmonary artery (ALCAPA) or double aortic arch. ALCAPA is a common defect on account of the left coronary artery anomalous origin from the pulmonary artery instead of the aorta. So there is often profound depression of myocardial function at the time of initial presentation. In the anastomosis operation of ALCAPA, a button-like aortic tissue was cut from ascending aorta and collected as a control sample. Double aortic arch is an abnormal formation of the aorta called as a vascular ring. Normally, the aorta is a single arch that leaves the heart and moves leftward. In double aortic arch, the ascending aorta divides into left anterior arch and right posterior arch, and these two aortic arches join posteriorly to form a vascular ring and further go around and press down on the windpipe and esophagus result in obstructive symptoms of them. In the operation of the double aortic arch, the smaller arches were dissected close to their junction with the descending aorta, and some aortic tissue was collected as the sample. All data collection complied with the regulations and was approved by the institutional ethics committees of Beijing Anzhen Hospital (Ref No. 2019051X). Clinical data of patients were collected from their medical records, echocardiographic records, and surgical records. The patients were informed of the purpose, procedures, and requirements. All participants provided informed written consent to take part in this study.

**Table 1:** Clinical characteristics of patients

Clinical characteristics	Sex	Age (month)	Body weight (kg)	Associated lesions
CoA group				
1	Male	3	6.3	PFO
2	Male	34	12	
3	Female	19	10	PFO
4	Male	5	8	
5	Male	32	11	
6	Male	10	8.5	
7	Male	8	6.1	PDA
8	Female	3	3.5	PFO
9	Male	22	12	
10	Female	6	6.5	PDA
11	Male	25	11	
12	Male	11	9.3	
Total	F/M(3/9)	14.8 ± 11.2	8.7 ± 2.7	
Control group				
1	Male	5	7	
2	Female	3	5.3	
3	Female	15	11	
4	Male	3	4	
5	Male	31	10	
6	Female	10	14	
7	Male	9	8.4	
8	Male	10	12.5	
9	Male	8	8.7	
10	Male	11	9	
Total	F/M(3/7)	10.5 ± 8.1	9.0 ± 3.1	
*P* Value	1.0	0.32	0.8	

*PDA* Patent ductus arteriosus, *PFO* Patent foramen ovale

### Limitations

This study had certain limitations. First, the sample size was small and might lead to bias. Second, the study was limited by specimen site restrictions. Owing to ethical reasons, tissues at the juxtaductal aorta could not be obtained. Also, the ascending aorta (sampled in patients with ALCAPA) and transverse aorta (sampled in patients with double aortic arch) might be different from normal juxtaductal aorta in the non-coarct patients.

### RNA isolation, library preparation, sequencing and bioinformatics analysis

All the excised vessel tissues were immediately put into liquid nitrogen for quick freezing, stored at -80 °C, and then RNA was extracted. Total RNA was extracted with Trizol (Tianjian, Beijing), the results were evaluated using Qubit Fluorometer (Invitrogen) and Agilent 2100 BioAnalyzer (Agilent Technologie, USA). The samples need to meet following requirements: 28S: 18S ratio > 1.8; RNA integrity index (RIN) > 7.0. We used CapitalBioTechnology (Beijing, China) to generate RNA-seq library and sequence, built an independent library with all measured samples (in triplicate) and performed sequencing and analysis. We used NEB Next Poly (A) mRNA Magnetic Separation Module kit gathering the poly (A) tail mRNA molecules. And the mRNA was broken into approximately 200 base pair fragments. The ends of the cDNA fragments were connected with adaptors after an end repair process. The products were enriched and purified through PCR. Quantitative analysis of final library was performed with the Agilent 2100 bioanalyzer and the KAPA library quantification kit. After verification by RT-qPCR, paired-end sequencing was performed on the library. And then we read 150 base pairs long on the Illumina HiSeq sequencer (Illumina) [10].

### RNA- sequencing data analysis

We used the human genome (hg19) as a reference. We used Fast QC to evaluate the quality of sequencing, and used NGSQC (v0.4) to filter low-quality data. We used HISAT2 (Johns Hopkins University) to set the default parameters to align clean roads to the reference genome [2]. We used HISAT2 (Johns Hopkins University) to compare the processed reads of each sample with the reference genome. Gene expression analysis was performed using Cuff Quant and Cuff norm (Cufflinks 2.2.1). We used the cuff difference to analyze the DEG among samples. Methods of standardization in Cuff diff are geometric, with each condition and summarized as discrete models [3]. Separately, we conducted thousands of independent statistics hypothesis tests on DEG, and obtained the corrected p-value through the false discovery rate (FDR) method. We calculated the corrected P value (q value) with the BH method for correction. Significant analysis was conducted to the P value or q value. The parameters used to significantly classify DEG were the twofold difference in transcription abundance (log^2^, FC: fold change in expression) and q < 0.05). The DEG’s annotation was according to the information obtained from the KEGG, ENSEMBL, NCBI, GO and Uniprot database.

### Real-time RT-PCR validation of RNA- sequencing

Vessel tissues were dissociated as describing above. The analysis of RT-PCR have been carried with ABI7500 (Applied Biosystems) with Super-Real PreMix Plus kit (SYBR Green). calponin 1-F: 5ʹ-ACACGGCGTCACCTCTATG-3ʹ; calponin 1-R: 5ʹ-TGAGTGTGTCGCAGTGTTCCA-3ʹ. MYH11-F: 5ʹ-CGCCAAGAGACTCGTCTGG-3ʹMYH11-R: 5ʹ-TCTTTCCCAACCGTGACCTTC-3ʹ. β-actin-F: 5ʹTCATGTTTGAGACCTTCAA-3ʹ. β-actin-R: 5ʹGTCTTTGCGGATGTCCACG-3ʹ was used as endogenous chain. All samples had been processed three times. Relative gene expression had been determined through ^ΔΔ^CT method.

### Western blotting assays

Tissue proteins of different group had been separated through 10% SDS–PAGE and the analysis Western blot was carried with different antibody such as anti-calponin (ABCOM ab46794), anti- MYH11 (ABCOM ab53219). The normal IgG of rabbit or mouse have been used as the negative control.

### Histology staining

We fixed vessel tissues in paraformaldehyde (4%) overnight (4 °C). We used paraffin to embeded them. And we used hematoxylin and eosin (HE) to stain the samples serial sections (6 μm) with for the analysis of histopathology. To determine elastic fibrosis the, the sections were stained with cudbear and Verhoeff’s Van Gieson. We used the previously described method to perform IHC staining for specific protein expression on vessel tissue sections. In short, the sections were incubated with primary antibodies against calponin (BA46794) Anti -MYH11 (AB53219) overnight at 4 °C. After rinsing in the PBS buffer, the samples were incubated with secondary antibody for 30 min. The control experiments included omitting the primary antibody and replacing the primary antibody with non-immunized rabbit, mouse or goat IgG. IHC staining results were tested through the Olympus BX51 microscope (Olympus, Japan). All of the measuring results were the average of three samples. All of the data were showed as meaning ± S.D. T test was used for statistical analysis.

### Electronic microscopy detection

We distributed the vessel tissues with 2% glutaraldehyde and 4% formaldehyde at a rate 3 mL/min in phosphate buffer (0.1 mol/L). Vessel tissues were put in the 3% glutaraldehyde solution at 4 °C overnight. After washing the tissue block with phosphate buffer (0.1 mol/L) in triplicate it was fixed in osmium tetroxide (1%) with phosphate buffer (0.1 mol/L) for 2 h at 4 °C. The samples were dehydrated and embedded. We stained ultra-thin sections of myocardial tissue with uranyl acetate and lead citrate, and then examined them with a transmission electron microscope.

## Results

### Histological features of the CoA in infants

**Fig. 2:**
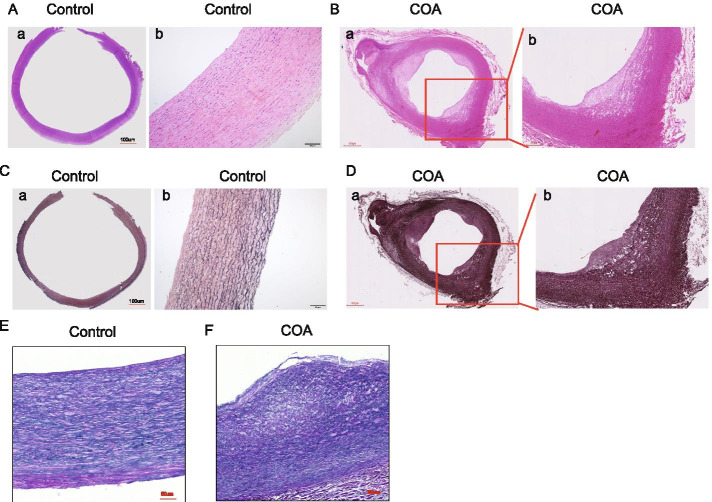
Histological features of control and CoA aortas. (A-a) Representative transverse section of control vascular tissues stained with hematoxylin and eosin (H&E). Bars: 100 μm. (A-b) Higher magnification of A-a. (B-a) Representative transverse section of CoA vascular tissues stained with H&E. Bars: 50 μm. (B-b) Higher magnification of the boxed areas from B-a. (C-a) Representative transverse section of control vascular tissues stained with cudbear. Bars: 100 μm. (C-b) Higher magnification of vascular tissues from C-a. (D-a) Representative transverse section of CoA vascular tissues stained with cudbear. Bars: 100 μm. (D-b) Higher magnification of the boxed areas from D-a. (E and F) Representative transverse sections of control and CoA vascular tissues stained with Verhoeff’s Van Gieson. Bars: 50 μm

The aortal narrowed segments obtained after the surgery of six children were tested using hematoxylin and eosin staining to characterize the histological changes in CoA in infants. The arterial intima in the CoA region was uneven and part of the arterial intima thickened compared with that in the control group (Fig. 2A-a and A-b). Extracellular matrix accumulated in the narrow part of the vessel shaped like a hill (we called the thickened intima as the inner membrane pad) (Fig. 2B-a and B-b). To determine elastic fibrosis, the sections were performed with cudbear and Verhoeff’s Van Gieson stain. Hematoxylin and Verhoeff’s Van Gieson staining suggested that elastic fibrosis was arranged neatly in control vascular tissues (Fig. 2C-a, C-b, and E). However, the internal and middle elastic laminae in CoA was disassembled and incomplete with the loss of elastic fibers in the medial layer or even disappeared compared with those in the control group (Fig. 2D-a, D-b, and F).

### RNA-seq identification of DEGs and GO analyses in the vascular tissues of patients with CoA

**Fig. 3:**
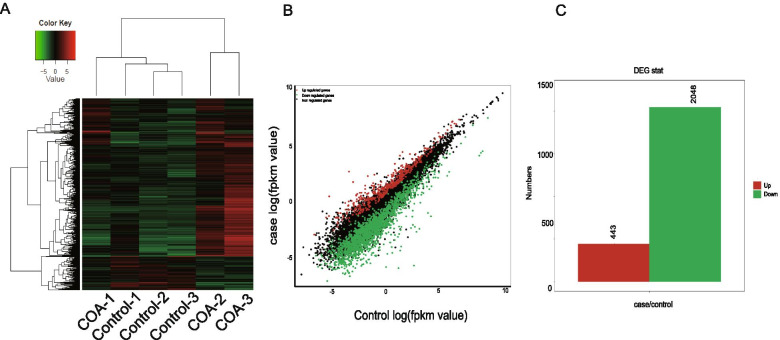
Differentially expressed genes in the RNA-Seq data. **A** Volcano plot of DEGs (*P* < 0.05) identified from the RNA-Seq libraries of control and CoA vessel tissues. **B** Scatter plot diagram of DEGs. The horizontal axis is control log(fpkm value). The case log(fpkm value) is plotted on the vertical axis. Each gene is represented by one point on the graph (red represents upregulation, and green represents downregulation). **C** Bar graph of DEGs (*P* < 0.05) identified from the RNA-Seq libraries of control and CoA vessel tissues

RNA-seq was performed in vascular tissues of patients with CoA and control patients to gain valuable insights into the mechanism of CoA. Illumina HiSeq 4000 was used to generate 55,803,812 raw reads, of which 51,513,550 reads were clean. The generation rate of clean readings for all samples was higher than 92.31%. Further, 39,691 differentially expressed mRNAs were identified between patients with CoA and normal controls (*P* adjusted value ≤ 0.05, fold change 2). Alignment statistics showed that the data had high quality and enough sequencing depth for differential expression. Also, 2491 DEGs were identified, which had a false discovery rate lower than 0.05, including 443 upregulated genes and 2048 down regulated genes (Fig. 3A-C).

### NHGRI-GWAS and KEGG analyses of DEGs

**Fig. 4:**
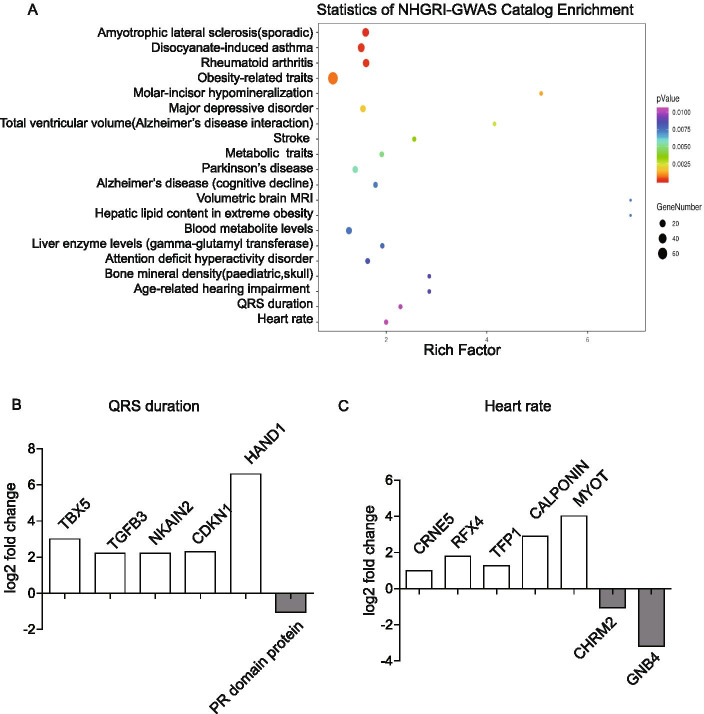
Statistics of NHGRI-GWAS Catalog Enrichment. **A** Statistics of NHGRI-GWAS Catalog Enrichment analysis of control and CoA vessel tissues. **B** Log^2^fold change of QRS duration– associated genes. **C** Log^2^fold change in heart rate– associated genes

**Fig. 5:**
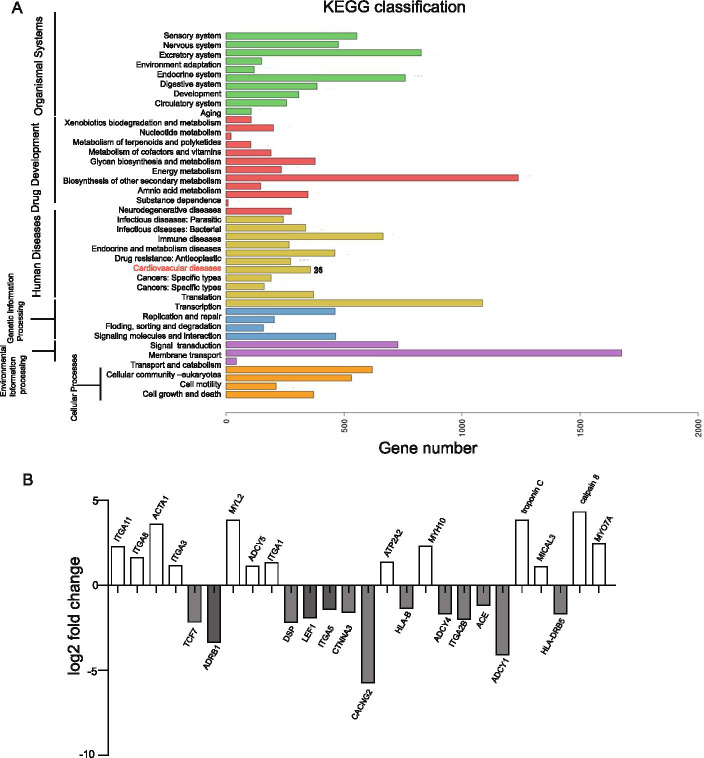
KEGG classification in the RNA-Seq data. **A** KEGG classification in the RNA-Seq data analysis of control and CoA vessel tissues. **B** Log^2^fold change in cardiovascular disease– associated genes

The National Human Genome Research Institute (NHGRI) genome-wide association studies (GWAS) Catalog and Kyoto Encyclopedia of Genes and Genomes (KEGG) analyses were used to better understanding the CoA gene networks. Further, 13 genes were found to be associated with QRS duration and heart rate change (Fig. 4A). These two indicators gained our attention because they were the characteristic symptoms of CoA. Among these 13 genes, 6 genes were associated with QRS duration and 7genes with heart rate. Of the six genes associated with QRS duration, five were upregulated and one was down regulated (Fig. 4B). Of the seven genes associated with heart rate, five were upregulated and two were down regulated (Fig. 4C). The KEGG analysis predicted that the DEGs were enriched in 44 pathways (Fig. 5A). Among all of the identified KEGG pathways, 26 genes were associated with cardiovascular disease, including 13 down regulated genes and 13 upregulated genes (Fig. 5B). DEGs mainly focused on extracellular matrix (ECM) organization, such as integrin-α 5–11(ITGA5-11), and muscle differentiation, including MYH10, MYH11, calponin, and α-actinin (Fig. 5B).

### Validation with qRT-PCR and IHC analysis

**Fig. 6:**
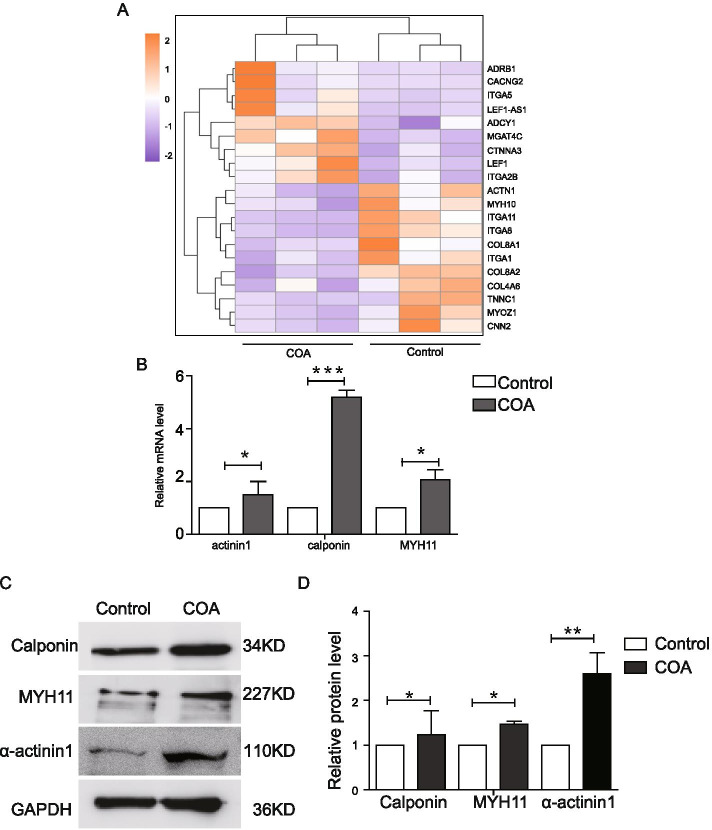
Validation analysis using quantitative RT-PCR, Western blot analysis, and IHC. **A** Volcano plot of DEGs associated with the development and differentiation of vascular SMCs (*P* < 0.05) identified from the RNA-Seq libraries of control and CoA vessel tissues. **B** qRT-PCR analysis of *actinin1*, *calponin *and *MYH11* in control and CoA vessel tissues. Real-time quantitative PCR was performed with gene-specific primers. The expression of each gene was normalized with the average expression of the endogenous reference gene β-actin. The error bar indicates the standard error of the mean fold change. **C** Total protein was extracted from control and CoA vascular tissues for Western blot analysis. **D** Protein bands were scanned, and relative band intensities were normalized

**Fig. 7:**
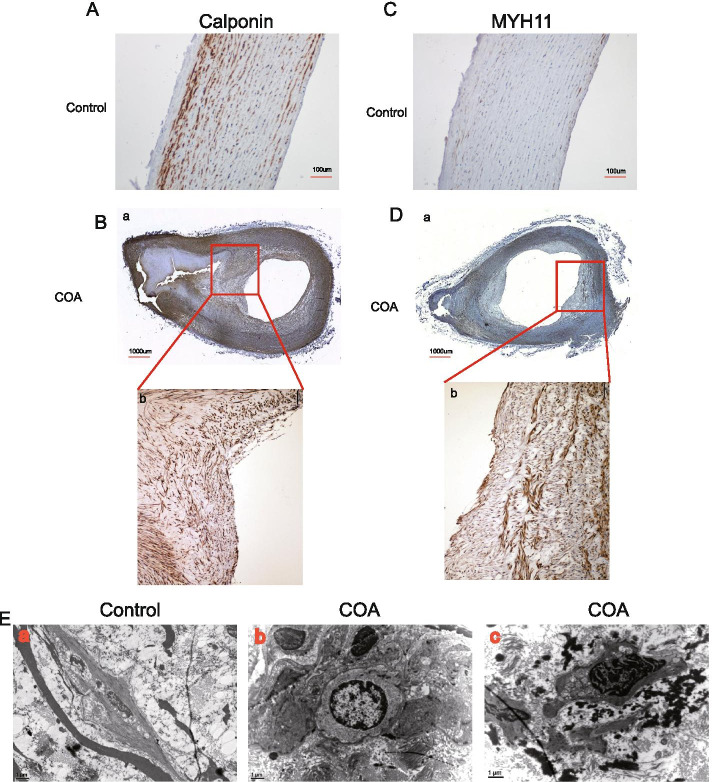
Validation analysis using IHC. (A) Immunohistochemical staining showed the level of calponin in control vessel tissues. (B-a) Immunohistochemical staining showed the level of calponin in CoAvascular tissues (upper scale bars: 20 μm). (B-b) Higher magnification of the boxed areas from the upper region. (C) Immunohistochemical staining showed the level of MYH11 in control vessel tissues. (D) Immunohistochemical staining showed the level of MYH11 in CoA vascular tissues (upper scale bars: 20 μm). (B-b) Higher magnification of the boxed areas from the upper region. (E) Representative images of transmission electron microscopy of the intima of control and CoA vascular tissues. (a) shows images of control intima. (b and c) show images of CoA intima. Magnifications have been indicated in each image

Hierarchical clustering analysis showed the expression profiles of the top 20 DEGs associated with the development and differentiation of vascular smooth muscle (Fig. 6A). Among the 13 upregulated genes, SMCs differentiation–associated genes, that is, MYH11, calponin and α-actinin1 were selected and confirmed by qRT-PCR (Fig. 6B) and Western blot analysis (Fig. 6C and D) in the vascular tissues of 6 patients with CoA and 4 control participants, which were not analyzed by RNA-seq. The IHC analysis was used to compare the protein expression levels of calponin and MYH11 in CoA and four control vascular tissues to explore the location of these makers. In the control group, calponin was mainly expressed on the base of the intima, and rarely expressed on tunica and adventitia media (Fig. 7A). In the coarctation, calponin had high expression in the whole-coarctation intima, especially in the thickening region of the intima (Fig. 7B). Also, MYH11 expression was higher in the thickening region of intima compared with that in the control group (Fig. 7C and D). The microscopic analysis was performed in vascular tissues of patients with CoA and control participants to detect further the ultrastructural structure in the intima. Electron microscopic analysis showed squamous endothelial cells and some elastic fibers in the control vessel intima (Fig. 7E-a). The dedifferentiated SMCs were mainly located on the surface of the inner membrane (Fig. 2E-b) and in the deep layers of the inner membrane pad in CoA (Fig. 7E-c). These dedifferentiated cells were characterized by hypertrophy with abundant myofilaments and a dramatic increase in the number of organelles.

## Discussion

CoA is a type of congenital heart illness rather than a kind of ordinary mechanical disease that can be treated completely by the intervention surgery alone [2]. Frequent recurrence of high blood pressure and atherosclerosis after surgical treatment hinders the assurance of patient’s quality of life [5]. Therefore, early diagnosis and appropriate treatment of infants with CoA are vital in reducing the mortality rate and preventing postoperative complications. However, indistinct pathology and molecular mechanisms of CoA have made this difficult. Novel findings of morphological and molecular biological research suggest that CoA is characterized by intimal thickening and impaired elastic fibers formation. These changes impair arterial elasticity. Two processes are involved in the formation of intimal thickening in CoA: (1) ECM accumulation in the subendothelial area; and (2) SMC migration of ductus arteriosus into inner media, resulting in internal elasticity of the frag-arteriosus [11]. Elzenga et al. detected that the coarctation shelf duct tissues were characterized by the loosely arranged spindle cells [12]. Although Elzenga et al. speculated that these spindle cells came from ECM, they did not characterize them. Irena Tanaskovic revealed some synthetic-phenotype SMCs on the shelf of CoA aortic wall [13]. These SMCs showed a fibroblast-like morphology, probably to proliferate and secrete extracellular matrix (ECM) components. These studies provided evidence that ECM and SMCs might participate in the pathogenesis of CoA. Despite these findings, the origin and phenotypic nature of SMCs in CoA are still elusive.

This was the first study using RNA-seq to identify DEGs in vascular tissues of patients with CoA mainly focusing on infants aged less than 3 years. A total of 2491 DEGs were detected. Among these DEGs, ECM-associated genes involved in contractile fiber and SMC differentiation were highly expressed in CoA samples. Recent findings demonstrated that SMCs in the media switch from a contractile state to a secretory state, become migratory, and travel to the intima, where they proliferate further to form neointimal lesionsin injuries of vessel function such as arteriosclerosis [14]. In this study, SMC differentiation–associated genes selected from DEGs, such as calponin and MYH11, were further validated with IHC staining and qRT-PCR to explore further the phenotype of SMC in the intimal thickening in COA. We found that calponin and MYH11 were highly expressed in CoA compared with control tissues (Fig. 6B). Calponin, as a marker of early stages of differentiation, was mainly expressed on the surface of the inner membrane pad (a hilly projection-like cushion formed by tissue proliferation in the inner membrane). MYH11, representing terminal differentiated SMCs was mainly located in deep layers of the inner membrane pad. This result implied that the SMCs do contribute to intima thickening. Regarding the source of these SMCs, it was speculated that one part of dedifferentiated SMCs invaded into the intima and destroyed elastic fibers of inner and middle layers, while the other part might bean extension of ductus into the wall of the aorta in CoA. To sum up, both parts of SMCs were involved in the thickening of the intima in infants with CoA. At the same time, electron microscopic analysis also showed dedifferentiated SMCs located in the thickening intima. This result was consistent with the theories proposed by other scholars. For example, Kim et al. found that the dedifferentiated SMC expression was in the intima of patients with CoA [15]. Jimenez examined the SMC phenotype of normal aortas, the coarcted shelf, and the moderately stenotic fields of the CoA. They also found that the dedifferentiated SMCs in CoA intima [16]. The pharyngeal arches and their arterial system contribute to the development of the aorta during the embryonic period [17]. The primary portion of the aortic arch develops from the fourth pharyngeal arch. Any deviation during this complex developmental process can lead to various aortic anomalies including CoA [17]. Existing studies pointed out that the tissues from the ductus arteriosus might get incorporated into the aortic wall where it was connected with the descending aorta during the development of the aortic arch. With the constriction of the ductus arteriosus after birth, the constriction in the isthmus area leads to the development of CoA [18]. However, the signaling pathway and molecules regulating this process are still unclear. Genetic studies suggest that CoA is associated with the regulation of Notch signaling, which contributes to the development of CoA [19]. Prostaglandin E receptor EP4, which is known to be a predominant prostanoid receptor in the ductus arteriosus, was reported to participate in ductal extension into the aortic wall in CoA [20]. In this study, a set of valuable genes were detected by RNA-seq and several genes associated with SMC differentiation were further identified to predict the development and progression of CoA, which might have important implications for the diagnosis and treatment of this disease. Further research into the cellular mechanisms involved in CoA might help understand the mechanisms of the aortic arch and also ensure that longer-term morbidity, secondary to early-onset hypertension and atherosclerotic progression may be minimized.

## Conclusions

Taken together, this study showed novel transcript expression differences between patients with CoA and control participants. Migration of medial smooth muscle cells (SMCs) or arterial duct SMCs to the subendothelial space was closely associated with the occurrence of CoA in infants. Our study provided insights into the mechanisms and novel biomarkers of CoA. Future challenges include not only to further investigate the validity of the hypothesis but also to characterize the phenotype of SMCs.

## Data Availability

All data used during the study are available from the corresponding author by request.
